# Role of SLC4 and SLC26 solute carriers during oxidative stress

**DOI:** 10.1111/apha.13796

**Published:** 2022-03-01

**Authors:** Alessia Remigante, Sara Spinelli, Michael Pusch, Antonio Sarikas, Rossana Morabito, Angela Marino, Silvia Dossena

**Affiliations:** ^1^ Biophysics Institute National Research Council Genova Italy; ^2^ 18980 Department of Chemical Biological, Pharmaceutical and Environmental Sciences University of Messina Messina Italy; ^3^ Institute of Pharmacology and Toxicology Paracelsus Medical University Salzburg Austria

**Keywords:** Cl^−^/HCO_3_^−^ exchangers, oxidative stress, oxidative stress‐related diseases, SLC26, SLC4

## Abstract

Bicarbonate is one of the major anions in mammalian tissues and fluids, is utilized by various exchangers to transport other ions and organic substrates across cell membranes and plays a critical role in cell and systemic pH homoeostasis. Chloride/bicarbonate (Cl^−^/HCO_3_^−^) exchangers are abundantly expressed in erythrocytes and epithelial cells and, as a consequence, are particularly exposed to oxidants in the systemic circulation and at the interface with the external environment. Here, we review the physiological functions and pathophysiological alterations of Cl^−^/HCO_3_^−^ exchangers belonging to the solute carriers SLC4 and SLC26 superfamilies in relation to oxidative stress. Particularly well studied is the impact of oxidative stress on the red blood cell SLC4A1/AE1 (Band 3 protein), of which the function seems to be directly affected by oxidative stress and possibly involves oxidation of the transporter itself or its interacting proteins, with detrimental consequences in oxidative stress‐related diseases including inflammation, metabolic dysfunctions and ageing. The effect of oxidative stress on SLC26 members was less extensively explored. Indirect evidence suggests that SLC26 transporters can be target as well as determinants of oxidative stress, especially when their expression is abolished or dysregulated.

## 1 INTRODUCTION

Oxidative stress (OS) is frequently described as an imbalance between the production of reactive oxygen species (ROS) in biological systems and the ability of the latter to defend themselves through their sophisticated antioxidant machinery.^1^, ^2^ The term ROS is applied to a collection of highly reactive chemicals, namely free radicals, including superoxide anion (O_2_^·−^), hydroxyl radical (OH^·^) and singlet oxygen (^1^O_2_), or alternatively their non‐radical intermediates, such as hydrogen peroxide (H_2_O_2_), nitric oxide (NO) and hypochlorous acid (HOCl), which are typically less reactive.^3^, ^4^ In eukaryotes, endogenous ROS represent a normal byproduct of the cell metabolism and mainly arise from the incomplete reduction of O_2_ during the process of oxidative phosphorylation in the mitochondria.^5^ In addition, endogenous ROS are produced by various cellular enzymes, including NADPH oxidases (NOXs) and nitric oxide synthase (NOS), peroxisomes, ionizing and UV radiation, as well as by the metabolism of drugs and xenobiotics.^6^, ^7^ However, the redox state in the cell is normally regulated by a complex endogenous antioxidant system, which is composed of proteins with enzymatic activities, like glutathione peroxidase, catalase (CAT), and superoxide dismutase and non‐enzymatic small‐molecule compounds, like glutathione, which are able to quickly neutralize ROS and ensure a low production of reactive species. In addition, antioxidant molecules, such as vitamin C, vitamin E, some minerals, carotenoids, and polyphenols, which can be supplied through the diet, can help the activity of endogenous antioxidants, thus promoting the redox homoeostasis of the cell.^8^

Nevertheless, some oxidants in controlled amount possess important cell defense and signalling functions within the cell. Specifically, cells can generate ROS with function of second messengers, use them for intracellular signalling and for stimulating redox‐sensitive pathways in order to modify the cellular levels of cytoprotective regulatory proteins.^9^, ^10^ For example, the massive ROS production by activated macrophages not only represents a first‐line defense against environmental pathogens, but can also stimulate T‐cell function, including the production of cytokines.^11^ However, when oxidants are produced in excess, or when the antioxidant defenses that regulate them are ineffective, the balance between antioxidant and pro‐oxidant capacity can be perturbed, thus resulting in OS and ultimately cell death through apoptosis or necrosis. In these conditions, biomolecules such as nucleic acids, membrane lipids, enzymes, and structural proteins can be altered through oxidation to an extent that exceeds repair capacity.^12^, ^13^ Abnormal ROS levels can influence several cell signalling pathways. In this regard, the role of OS in the pathogenesis of disease is widely acknowledged. A perturbed redox homoeostasis may be the common denominator underlying ageing and different chronic diseases,^14^, ^15^, ^16^, ^17^, ^18^, ^19^ although specific mechanisms contributing to OS‐induced damage are poorly investigated.

By representing the boundary between the cell interior and the extracellular environment, the cell membrane is most vulnerable to free radical attack.^20^ The plasma membrane contains a wide range of proteins and lipids that elicit distinct cellular reactions in response to extracellular stimuli and stressors, and each of these can be target of OS. The solute carrier (SLC) superfamily of transporters comprises integral membrane proteins known as the gatekeepers for all cells, as they control the transmembrane flux of inorganic ions, sugars, amino acids, nucleotides, fatty acids, neurotransmitters, and drugs.^21^ Currently, the human SLC superfamily includes over 458 members grouped into 65 families.^22^ This organization has been established by the Gene Nomenclature Committee (HGNC) of the Human Genome Organization (HUGO), and arranged such that member proteins within each family share at least 20%‐25% sequence similarity with at least one other member of the family.^23^ The SLC transporters are grouped into four main types according to the transport mode, which are cotransporters, exchangers, facilitated transporters and orphan transporters (with no known substrate), thus playing a central role in a plethora of physiological and pathological functions in almost all cells and tissues.^22^ These membrane transporters are widely expressed throughout the body, most notably in the epithelia of major organs, such as the liver, intestine, kidney and organs with barrier function, such as the blood‐brain barrier, testes and placenta. Many transporters are also expressed in an organ‐specific manner, thus facilitating the entry and elimination of endogenous and xenobiotic compounds.^24^ The substrate specificity of these transporters can be determined by interactions between the amino acid backbone and/or side chains and the substrate, as well as by intramolecular interactions that regulate gating and/or selectivity elements.^25^

The modulation of the activity of membrane transport systems is part of the cellular response to OS.^26^ In particular, the link between OS and membrane transport systems during ageing as well as in OS‐related diseases is incompletely understood, and is essential for a deeper understanding of mechanisms through which such processes and diseases develop. Indeed, the relationship between ion transport and cellular redox balance is complex. The activity of ion transporters and channels can be stimulated or blocked during OS and, in turn, these molecular entities can even be involved in determining or attenuating OS. To name just a few examples, volume regulated anion channels LRRC8/VRAC can be either activated or inhibited by OS depending on their subunit composition, while LRRC8/VRAC inhibitors seem to lower OS.^27^ The expression as well as the function of the cystic fibrosis transmembrane conductance regulator (CFTR) chloride channel is impaired during OS, thus contributing to the progression of airway dysfunctions, including chronic obstructive pulmonary disease (COPD), consequent to alteration of the mucociliary transport in airway epithelial cells.^28^ On the other hand, CFTR is permeable to not only chloride ions, but also organic anions such as reduced glutathione, and consequently CFTR dysfunction potently contributes to the OS burden at the airway surface in cystic fibrosis.^29^ Recent studies have established that the Na^+^/K^+^‐ATPase can cause OS with mechanisms distinct from its well‐understood function of ion pump but rather dependent on the scaffolding properties of the alpha1 subunit, which is targeted by OS and post‐translationally modified, thus leading to the activation of a downstream signalling cascade eventually amplifying ROS production.^30^

In this review, we will focus on the impact of OS on plasma membrane SLC transporters, and specifically on the members of SLC4 and SLC26 families of chloride/bicarbonate (Cl^−^/HCO_3_^−^) exchangers, which are essential for maintaining crucial homoeostatic functions, such as the regulation of systemic and intracellular pH and ion composition, and the regulation of cell and extracellular fluid volume.^31^, ^32^ We will emphasize their pathophysiological relevance and their role in OS‐related conditions, including ageing. In this regard, only isoforms involved in OS events will be considered. Also, we will discuss their potential as targets of antioxidant therapies.

## 2 THE SLC4 FAMILY

The SLC4 transporters, also known as the bicarbonate‐transporter family, are integral membrane proteins that carry bicarbonate (HCO_3_^−^) and other electrolytes (Na^+^ and Cl^−^) across the plasma membrane. These proteins play a critical role in the acid‐base homoeostasis of the body by acting as either acid loaders or acid extruders, thus regulating both intra‐ and extracellular pH.^33^ Based on the transport modes, members of the SLC4 family of proteins were classified into three functional groups in mammals: (1) Na^+^‐independent Cl^−^/HCO_3_^−^ exchangers, which include SLC4A1, SLC4A2, and SLC4A3; (2) Na^+^‐dependent HCO_3_^−^ transporters, comprising electrogenic Na^+^/HCO_3_^−^ cotransporters (SLC4A4, SLC4A5), electroneutral Na^+^/HCO_3_^−^ cotransporters (SLC4A7 – formerly SLC4A6 – and SLC4A10), and an electroneutral Na^+^‐driven Cl^−^/HCO_3_^−^ exchanger (SLC4A8); (3) Na^+^‐coupled borate transporter that do not transport bicarbonate, which is represented by the unique member SLC4A11.^34^ The classification of SLC4A9 remains unclear because its function is still incompletely characterized.^35^

The Na^+^‐independent Cl^−^/HCO_3_^−^ exchangers, and particularly SLC4A1 and SLC4A2, have been well characterized in the context of OS and will be reviewed in the following. For the other SLC4 family members, there is no information available (Table 1).

**TABLE 1:** Link between SLC4A members and oxidative stress (OS)

Transport mode	Isoform	Tissue distribution	Experimental model	Findings	References
Na^+^‐independent Cl^−^/HCO_3_^−^ exchangers	SLC4A1	Erythrocytes, kidney, heart, colon	Erythrocytes exposed to H_2_O_2_	Reduction of transport efficiency (SO_4_^2−^)	62, 98
			Erythrocytes exposed to thiol‐oxidizing agents	Reduction of transport efficiency (SO_4_^2−^) Oxidation of membrane SH groups	64, 97
			Erythrocytes exposed to extracellular pH variations	Reduction of transport efficiency (SO_4_^2−^) Oxidation of membrane SH groups	102, 103
			Inflammation‐associated diseases	Acceleration of transport efficiency (SO_4_^2−^) Tyrosine phosphorylation increase Lipid peroxidation increase	67, 68, 71
			Canine Leishmaniasis	Reduction of transport efficiency (SO_4_^2−^) Lipid peroxidation increase Protein degradation	74, 75, 76
			Diabetes mellitus	Acceleration of transport efficiency (SO_4_^2−^) Oxidation of membrane SH groups Lipid peroxidation increase Reduction of GSH/GSSG ratio Protein degradation	80
			Erythrocytes exposed to high glucose	Acceleration of transport efficiency (SO_4_^2−^) Reduction of GSH:GSSG ratio	80
			Erythrocytes exposed to 0.1‐10 mmol/L D‐Gal	Reduction of transport efficiency (SO_4_^2−^) Formation of glycated haemoglobin	85
			Erythrocytes exposed to 100 mmol/L D‐Gal	Acceleration of transport efficiency (SO_4_^2−^) Lipid peroxidation increase Reduction of GSH:GSSG ratio Oxidation of membrane SH groups Formation of Methaemoglobin Formation of glycated haemoglobin	93
			Age‐related diseases	Protein degradation	87, 89, 91
	SLC4A2	Kidney, gut, blood vessels, lung	Endothelial cells exposed to high glucose	Increase of protein expression Apoptosis	111
			Rat airway epithelial cells exposed to H_2_O_2_	Increase of protein expression Increase of transport efficiency (O_2_^·^‐)	112
	SLC4A3	Brain, heart, retina, pituitary, adrenal gland	No information available		
Na^+^‐dependent HCO_3_^−^ transporters	SLC4A4	Kidney, eye, brain, pancreas, heart	No information available		
	SLC4A5	Liver, kidney skeletal muscle	No information available		
	SLC4A7	Brain, testes, kidney, ovary	No information available		
	SLC4A8	Brain, testes, kidney, ovary	No information available		
	SLC4A10	Brain, heart, kidney, uterus	No information available		
	SLC4A9	Unclear	No information available		
Na^+^‐coupled borate transporters	SLC4A11	Kidney, salivary glands, testis, thyroid, trachea	No information available		

[Correction added on March 30, 2022 after first online publication. The duplicate row for Isoform SLCA4 was removed.]

### 2.1 The SLC4A1 isoform

The SLC4A1 isoform, also known as anion exchanger 1 (AE1) or band 3 protein (B3p), is encoded by the *SLC4A1* gene and—with more than 1 million copies per cell—is the most abundant membrane protein in human erythrocytes.^36^ A truncated form of SLC4A1 lacking the first 65 residues is expressed on the basolateral membrane of the alpha intercalated cells of the distal portion of the nephron, where it plays a crucial role in the reabsorption of bicarbonate, and consequently in urinary acid secretion.^37^ Some forms of distal renal tubular acidosis are caused by inherited mutations in the *SLC4A1* gene.^38^

**FIGURE 1:**
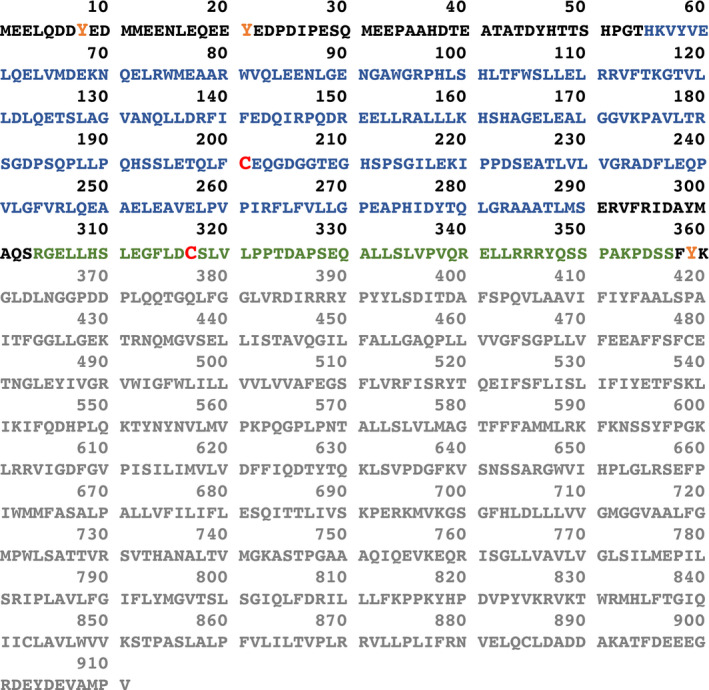
Amino acid sequence of human red blood cells B3p. Human erythrocyte B3p is a 110‐kD glycoprotein comprising a cytosolic N‐terminal domain (residues 1‐360) and an integral membrane domain (residues 361‐911, shown in grey).^39^ The globular domain spanning residues 55‐290 are shown as blue letters and the dimerization arm residues 304‐357 are shown in green. Cysteines that can form an inter‐subunit disulphide bond upon treatment with oxidizing agents (C201 and C317) are shown as red letters.^40^, ^61^ Phosphorylatable thyrosines (T8, T21 and T359) are shown in orange. NCBI Reference Sequence: NP_000333.1, single letter amino acid code

In 2015, the crystal structure of B3p revealed two domains: an N‐terminal cytosolic domain that anchors the cytoskeleton at the membrane and interacts with different erythrocyte proteins and a C‐terminal membrane domain that mediates the anion exchange^39^ (Figure 1). Moreover, B3p is present as a mixture of dimers and tetramers in membranes and these oligomers form interaction hubs around which integral and peripheral membrane proteins are organized.^40^ As an integral membrane protein, B3p is also important for the mechanical properties of red blood cells, such as docking of glycolytic enzymes and maintenance of cell shape by anchoring the actin‐spectrin cytoskeleton at the plasma membrane.^41^ Defects or deficiencies in B3p may lead to a reduction of cohesion between the cytoskeleton and the lipid bilayer, with a consequent loss of membrane surface area typical of hereditary spherocytosis.^42^

In physiological conditions, B3p mediates electroneutral Cl^−^/HCO_3_^−^ exchange across the plasma membrane, a fundamental process for an efficient respiration.^43^ Specifically, in peripheral tissues, carbon dioxide, which is generated by metabolic processes, diffuses into the erythrocytes and is hydrated by intracellular carbonic anhydrase II (CAII) to produce bicarbonate, which is in turn transported out of the cell in exchange for chloride.^43^ Conversely, at the level of pulmonary capillaries, the mechanism is reversed: HCO_3_^−^ enters the erythrocytes *via* B3p in exchange for Cl^−^ and is converted by CAII to carbon dioxide, which then diffuses across the plasma membrane to be excreted by the lungs.^44^ In this way, more than two thirds of the carbon dioxide molecules are transported in the form of HCO_3_^−^. Each B3p molecule exchanges 10^5^ pairs of monovalent anions per second. The Cl^−^/HCO_3_^−^ exchange is the largest ion‐specific flux of a secondary active transporter known in the body,^45^ and it is blocked by the unspecific anion exchange inhibitor 4,4‐diisothiocyanatodihydro‐stilbene‐ 2,2‐disulphonic acid (DIDS) with high affinity.^46^

**FIGURE 2:**
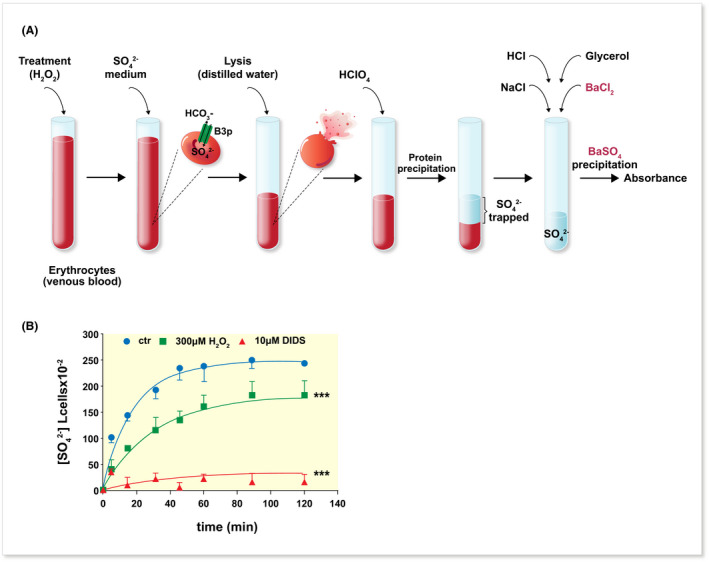
Turbidimetric method to measure the ion transport activity of SLC4A1/Band 3. (a) Erythrocytes are treated as required by the experimental protocol, resuspended in a sulphate‐rich buffer and osmotically lysed after established incubation intervals to determine the kinetics of sulphate uptake. After precipitation of proteins with HClO_4_, the amount of sulphate trapped by erythrocytes is determined spectrophotometrically following precipitation of BaSO_4_. (b) Kinetics of sulphate uptake in human erythrocytes treated for 30 min with 300 μmol/L H_2_O_2_, 10 μmol/L DIDS or left untreated (control, ctr). ***P < .001 vs control, one‐way ANOVA followed by Bonferroni's post hoc test. DIDS, 4,4′‐Diisothiocyano‐2,2′‐stilbenedisulphonic acid. Modified from Ref.^62^ [Correction added on March 30, 2022 after first online publication. The lablels A and B have been added to the figure in this version.]

In experimental conditions, the functional efficiency of B3p can in principle be estimated by measuring the influx or efflux of radiolabelled substrates across the plasma membrane or variations in the intracellular pH and chloride concentration, which can be revealed by sensitive dyes. However, the Cl^−^/HCO_3_^−^ exchange is so fast that it cannot be easily monitored without the confounding factor of the cellular metabolism,^44^ and sophisticated instrumentation is required to follow the time course of extracellular pH.^47^ In this respect, B3p transporter can exchange different anions, including SO_4_^2−^, even if at different rates. The use of SO_4_^2−^ transport to monitor B3p activity offers the advantage that the exchange time is slow enough to employ relatively simple experimental protocols.^48^ Moreover, the absence of SO_4_^2−^ within the erythrocyte ensures that intracellular SO_4_^2−^ determinations are essentially indicative of the anion uptake. This experimental approach has been recognized as an effective tool to monitor erythrocyte homoeostasis in several experimental conditions in vitro and ex vivo.^49^ In this regard, the rate constant for SO_4_^2−^ transport can be measured by using a turbidimetric method aimed at quantifying the amount of SO_4_^2−^ internalized through B3p as a function of time (Figure 2).

#### 2.1.1 SLC4A1 and oxidative stress

**FIGURE 3:**
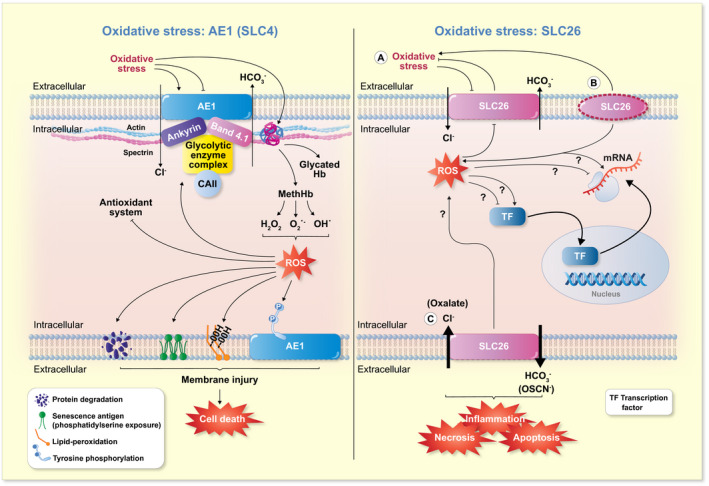
SLC4 and SLC26 family members and oxidative stress. Left: In human erythrocytes, increases in OS modify the physiological activity of SLC4A1 (AE1), an effect that translates into either stimulation or inhibition of the ion transport efficiency depending on the nature of the oxidant stimulus and concomitant pathological conditions. OS also affects the binding between AE1 and spectrin *via* the ankyrin bridge and/or glycolytic enzyme complex, as well as the interaction between AE1 and Hb. Moreover, elevation of ROS levels suppresses the intracellular antioxidant system and induces the formation of glycated Hb and MethHb. Possible cellular damages induced by increases in OS are (i) AE1 protein degradation, (ii) cell senescence, (iii) lipid peroxidation and (iv) tyrosine phosphorylation. Right: In epithelial cells, a normally functioning SLC26 exchanger (a) is protective against OS but, in turn, can be targeted by OS. In the context of OS, changes in the transcript levels of SLC26 family members have been reported, but the corresponding mechanisms have not been explored. Dysfunction (b) or hyperfunction (c) of SLC26 members have both been described in association with increased OS. AE1, anion exchanger 1 (Band 3 protein); CAII, carbonic anhydrase II; Hb, Haemoglobin; MethHb, Metahaemoglobin; ROS, reactive oxygen species

Erythrocytes are continuously threatened by oxidative events associated with high ROS levels in the blood stream and are therefore more exposed to OS than other cells. Indeed, erythrocytes are a prime target for oxidative stress due to their main function as O_2_‐carrying cells and may accumulate oxidative damage when crossing a tissue with an intense production of reactive species.^50^, ^51^ Moreover, OS in blood might rise following exposure to xenobiotics, auto‐oxidation of haemoglobin or release of ROS from neutrophils and macrophages into the plasma.^52^, ^53^ Thus, erythrocytes have sophisticated antioxidant defense machinery, and these aspects render these cells a good model for OS‐related studies.^54^, ^55^ In fact, the oxidants can exert their effects on the plasma membrane with potential consequences on transport systems and, in turn, on erythrocyte homoeostasis, which is strictly linked to B3p function. The transporter is particularly susceptible to redox balance variations in red blood cells and reductions in the efficiency of the antioxidant machinery, which encourage the capability of oxidizing molecules to generate ROS, thus resulting in intracellular cytotoxic injury^56^ (Figure 3).

A large body of evidence supports the notion that OS induces post‐translational modifications of the N‐terminus of B3p, which is exposed to the intracellular environment (Figure 1). Oxidized B3p is selectively and abundantly phosphorylated, which can induce B3p aggregation, weaken the interaction with the spectrin‐actin cytoskeleton and reduce membrane stability. Lyn is responsible for the phosphorylation of Tyr 359, and Syk is responsible for the phosphorylation of Tyr 8 and Tyr 21.^57^, ^58^, ^59^ In addition, oxidation induces B3p disulphide cross‐linking. The dimerization of B3p is guided by formation of disulphide bridges between two cysteine (Cys) residues located in the dimerization arms of the two monomers. The Cys 201 residue in one monomer and the Cys 317 residue of the paired monomer can easily form intermolecular disulphide bonds following moderate OS, which might block ankyrin binding.^40^, ^60^, ^61^ It is likely to hypothesize that these modifications might induce conformational changes that could affect B3p ion transport efficiency.

B3p transport efficiency has been firstly investigated in human red blood cells exposed to hydrogen peroxide (H_2_O_2_). This compound, which is commonly used in in vitro assays to model oxidant conditions, easily permeates the plasma membrane. In this regard, it has been demonstrated that not haemolytic concentrations of H_2_O_2_ induce OS and reduce B3p transport efficiency (Figure 2).^62^ In addition, it has also been reported that the anion transport by B3p can be modulated by oxygen pressure levels in human erythrocytes, being higher with higher oxygen pressure. Taking into account that haemoglobin binds B3p, these findings suggest that the transition of haemoglobin from the deoxygenated to the oxygenated form may play a key role in the regulation of anion exchanger activity.^63^ Moreover, OS induced by thiol‐oxidizing agents, such as N‐ethylmaleimide (NEM) or diamide, impaired the ability of B3p to drive the uptake of SO_4_^2−^ in human erythrocytes. The decreased efficiency of anion transport was linked to changes in the structural state of B3p caused by membrane sulphydryl groups oxidation, mainly belonging to B3p.^64^ In addition, these agents are known to cause B3p cross‐linking, thus inducing conformational changes, which in turn could affect the uptake kinetics.

In the last few years, erythrocytes have emerged as the main determinant of blood rheology.^65^ As mentioned above, B3p provides an anchoring point between the cell membrane and the cytoskeleton, which are the two main cellular structures that contribute to erythrocyte deformability. Human erythrocytes are capable of extreme changes in shape. Due to their flexibility, red blood cells can easily be compressed to pass through capillaries and can recover rapidly to their original shape. Therefore, potential defects in the integrity of the structure can produce changes in erythrocyte deformability and stability, affecting cell survival, homoeostasis, and rheological properties of blood. Such abnormalities are seen in different pathologies. In this respect, B3p has been studied ex vivo in some OS‐related diseases (Table 1).

#### 2.1.2 SLC4A1 and oxidative stress in inflammation

Several studies explored how OS‐linked modifications of B3p contribute to the development of inflammatory diseases. In general, red blood cells respond to OS by activating tyrosine kinases that induce tyrosine phosphorylation (Tyr‐P) at the cytoplasmic domain of B3p, thus leading to erythrocyte membrane destabilization.^66^ Thus, in normal red blood cells, Tyr‐P levels of B3p are closely controlled, but can be altered in pathological conditions. In fact, it has been shown that B3p Tyr‐P levels are useful in analyzing erythrocyte membrane functional status and are increased in inflammatory processes associated with systemic OS, such as endometriosis.^67^ In addition, the increased B3p Tyr‐P levels seen in G6PD deficiency correlated closely with chronic impairment of antioxidant defenses,^68^ whereas the lower B3p Tyr‐P levels observed in pregnancy are accompanied by a characteristically increased antioxidant defense.^69^

A large number of inflammatory mediators, including the C‐reactive protein (CRP), have been proposed as potential markers of inflammatory response.^70^ In this respect, Morabito and collaborators^71^ have investigated the effect of high serum CRP levels, associated to acute inflammatory diseases of various origins, on anion exchange capability through B3p in human erythrocytes. In this study, the anion exchange rate was accelerated in erythrocytes from patients with high (>8 mg/L) CRP levels. Though CRP levels returned to normal after 1 week, a total restoration of the rate constant for anion exchange was only observed after 2 months, suggesting that erythrocytes function was irreversibly affected and a total erythrocyte turnover was needed to normalize B3p functionality. Lipid peroxidation, which is associated to inflammation, correlated to erythrocytes deformability^72^ and could explain the accelerated uptake *via* B3p. Thus, these studies suggest a link between inflammation and B3p functional and/or structural alterations.

Among inflammatory conditions, Canine leishmaniasis is also associated with ROS generation and a reduction of erythrocyte survival. The leishmaniasis parasite, even though not directly invading red blood cells, causes a decrease in cell membrane fluidity, as well as an increase in cell rigidity, which may account for the reduced B3p transport efficiency observed in erythrocytes from infected dogs.^73^, ^74^, ^75^ This hypothesis is supported by the significant shape alterations unrelated to a decrease in cell size detected in erythrocytes from infected animals, suggesting a reorganization of membrane structure. In this regard, both an increase of lipid peroxidation in erythrocytes of infected animals and a marked degradation of membrane proteins, namely B3p and Band 4.1 protein, have also been demonstrated.^76^

#### 2.1.3 SLC4A1 and oxidative stress in metabolic dysfunctions

Diabetes mellitus is a chronic metabolic disorder characterized by insulin deficiency, insulin insensitivity, or both, as well as by hyperglycemia and vascular complications. Oxidative stress is a major participant in the pathophysiology of diabetes and not only promotes the onset of diabetes but also exacerbates its associated complications.^77^ This condition has been reported to dramatically impact on red blood cells, inducing membrane destruction, decreased deformability, alterations in haemoglobin oxygen binding and modification of the internal structure between membrane and cytoskeleton, thus leading to an altered systemic homoeostasis.^78^

To investigate the possible influence of diabetes on B3p transport efficiency, two different conditions have been considered: erythrocytes from diabetic patients and from healthy volunteers exposed to increasing concentrations of glucose for 24 hours in vitro, according to.^79^ The anion exchange rate *via* B3p was accelerated in both conditions.^80^ Specifically, in the first case, the functional alteration was linked to a modification of B3p conformation, putatively caused by an altered cross linking with haemoglobin, which is glycated in diabetic patients (6.5% or higher in this study), along with an increased OS, as revealed by an increased lipid peroxidation as well as decreased membrane sulphydryl groups abundance and GSH:GSSG ratio. Accordingly, it has been shown that hyperglycaemia may favour the advanced glycation end products formation, of which receptors activate signal transduction pathways, thus inducing ROS production.^81^ On the contrary, in the second case, although no significant levels of glycated haemoglobin and lipid peroxidation were detected and membrane sulphydryl groups abundance was not altered, a decreased GSH:GSSG ratio was found, which was normalized by pre‐exposure to the antioxidant melatonin. These findings suggest that alterations in B3p function may reveal early diabetic changes linked to OS.^80^

Galactosemia is a group of disorders of galactose metabolism characterized by erythrocyte galactose‐1‐phosphate levels usually >10 mg/dL. If undiagnosed or left untreated, galactosemia can result in life‐threatening complications including failure to thrive, hepatocellular damage, bleeding, sepsis, and neonatal death. Despite adequate treatment, children with galactosemia remain at increased risk for developmental delays, apraxia of speech, abnormalities of motor function, and hypergonadotropic hypogonadism or premature ovarian insufficiency in females.^82^ OS plays a fundamental role in the pathophysiology of galactosemia, which is also characterized by the formation of glycated haemoglobin.^82^, ^83^, ^84^ Remigante et al^85^ have reported that the rate constant for SO_4_^2−^ uptake *via* B3p is significantly reduced in erythrocytes treated for 1 hour with 0.1‐10 mmol/L D‐Galactose (D‐Gal). This effect was accompanied by the formation of glycated haemoglobin rather than OS, which was mitigated by the endogenous catalase. These findings suggest that B3p dysfunction might contribute to the pathophysiology of galactosemia and further underscore the sensitivity of B3p function for the formation of glycated haemoglobin.

#### 2.1.4 SLC4A1 and oxidative stress during ageing

Oxidative stress has also been postulated to play an important role in pathophysiological pathways involved in ageing as well as several age‐related diseases. The plasma membrane of red blood cells plays a crucial role in regulating cellular homoeostasis, and proteins and lipids involved in this function are very susceptible to oxidative modifications during ageing.^86^ In fact, proteomic studies have reported that 15% of erythrocytes in patients with Alzheimer´s disease (AD) are elongated and have an altered membrane architecture,^87^ which favours erythrocyte aggregation and sedimentation in the blood flow. In this regard, the removal of senescent erythrocytes in AD implies conformational changes in B3p, which are involved in both an increase of cell density and reduction in cell volume.^88^ In addition, in Alzheimer subjects, an accelerated breakdown of B3p has been shown,^89^, ^90^, ^91^ in line with what has been recently demonstrated in diabetic patients.^80^

Unfortunately, no studies exploring possible functional changes of B3p in aged individuals are reported in the literature. Chronic administration of D‐Gal has been widely used as a model to mimic a process very similar to the natural ageing, provoking OS *via* increased production of ROS and changes in antioxidant enzyme activities in the cell.^92^ In human erythrocytes exposed to 25‐100 mmol/L D‐Gal for 24 hours, the rate constant of SO_4_^2−^ uptake *via* B3p was increased; however, the total amount of SO_4_^2−^ trapped by the cell was significantly reduced. These changes in the transport properties were paralleled by and increased formation of glycated haemoglobin, as well as a marked OS.^93^ These findings confirm that the biophysical properties of B3p are sensitive to the oxidative status of the cell as well as to the formation of glycated haemoglobin and further suggest that B3p dysfunction might participate in age‐related pathophysiological alterations.

#### 2.1.5 SLC4A1 and beneficial effects of antioxidants

Both the enzymatic and non‐enzymatic antioxidant system are essential for the cellular response in order to deal with OS injuries. Also, antioxidants provided by the diet help the activity of endogenous antioxidants and contribute to the maintaining of redox homoeostasis.^94^, ^95^ Magnesium (Mg^2+^) deficiency has been associated with an increased production of ROS, increased levels of inflammation, and several age‐related diseases.^96^ G6PDH‐deficient red blood cells, which are exposed to increased OS levels compared with normal red blood cells, exhibited a decreased sulphate uptake *via* B3p. Magnesium pre‐treatment ameliorated the efficiency of anion exchange in these cells.^64^ To better investigate the mechanism of the antioxidant effect of Mg^2+^, the phosphorylation grade of B3p and tyrosine kinase Syk expression have been assayed in normal erythrocytes after OS induced by exposure to NEM.^97^ This investigation has shown that the beneficial effect of Mg^2+^ is not mediated by phosphorylation pathways, but is rather linked to an improvement of the endogenous antioxidant system and protection of SH groups. In fact, pre‐exposure to Mg^2+^ restored B3p ion transport following an increase of intracellular glutathione levels.^97^ Furthermore, the reduction of B3p anion exchange efficiency caused by a strong OS (300 μmol/L H_2_O_2_) could be prevented or attenuated by a short‐time pre‐incubation of red blood cells with low (10 μmol/L) H_2_O_2_ concentrations.^98^ This pre‐incubation encourages erythrocytes to adapt to a mild and transient OS and favours an increased tolerance to a successive stronger oxidant condition. Such adaptation response, termed preconditioning, could be monitored through B3p functional measurement, did not involve B3p‐related Tyr‐P pathways but was mediated by an increased activity of catalase. In fact, this strategy allowed red blood cells to optimize the performance of the endogenous antioxidant system, thus preventing the generation and accumulation of ROS and providing a better protection against oxidative damage.^99^

In a recent study, a correlation between melatonin effect, OS injury and B3p anion exchange capability was shown. In particular, the authors reported that pre‐treatment of human erythrocytes with melatonin ameliorated the reduction in rate constant for SO_4_^2−^ uptake *via* B3p, as well as the reduction in B3p expression levels was observed following treatment with H_2_O_2_.^100^ In parallel, exposure of erythrocytes to melatonin also prevented the increase in the rate constant for SO_4_^2−^ uptake observed in a cell‐based model of hyperglycaemia represented by red blood cells treated with high glucose concentrations (15‐35 mmol/L) for 24 hours.^80^

Apart from haemolysis, exposure of red blood cells to low pH values can evoke alterations of cell membranes.^101^ In fact, erythrocytes, when exposed to an external medium of different pH values, may exhibit alterations of cytoskeletal and integral membrane proteins, including B3p, which result in membrane destabilization and ionic imbalance probably caused by oxidative damage.^102^ Furthermore, a significant reduction in anion exchange efficiency has been detected, probably due to oxidized haemoglobin, resulting in damage to the cell membrane.^103^ According to this model, perturbations of the external medium may reflect on B3p transport efficiency, which critically depends on the interaction with intracellular proteins, such as haemoglobin. In this regard, curcumin protected erythrocyte membranes against OS associated to extracellular pH variations by scavenging free radicals.^102^

B3p is expressed in all cells and tissue of the body, including brain and lymphocytes, and represents an excellent marker protein for post‐translational modifications during ageing. In this respect, the anion transport ability of B3p decreased in brains and erythrocytes from old mice and, in addition, this functional reduction was associated to obvious structural changes.^104^ In parallel, the anion transport by lymphocytes also declined with age and these alterations could be delayed or prevented by increased levels of vitamin E supplied by the diet. Thus, vitamin E, which acts at membranes level, where the transporter resides, restored the anion exchange *via* B3p.

### 2.2 The SLC4A2 isoform

SLC4A2, or anion exchanger 2 (AE2), is an electroneutral Cl^−^/HCO_3_^−^ exchanger that in humans is encoded by the *SLC4A2* gene. This transporter is expressed in many tissues, including blood vessels, airways epithelia, proximal colon, and salivary glands cells and regulates the intracellular pH and Cl^−^ concentration.^31^, ^105^ In addition to these regulatory functions, AE2 exerts a central role in the transport of superoxide radicals,^106^, ^107^ and is also activated by low concentrations of NH_4_^+^ in several cell types. All AE anion exchangers have a large N‐terminal cytoplasmic domain, which is about 700 amino acid long for AE2, followed by a 500 amino acid polytopic transmembrane domain and a short C‐terminal cytoplasmic tail.^108^ Crosslinking studies in gastric parietal cell membranes suggest that AE2 possesses a dimeric structure.^109^

#### 2.2.1 SLC4A2 and oxidative stress

Diabetes mellitus causes a wide variety of vascular complications that are closely related to the degree of glycaemic control, suggesting that abnormal blood glucose levels are a critical risk factor for the damage of endothelial cells.^110^ In a model of hyperglycaemia based on human umbilical vein endothelial cells (HUVECs), high concentrations of glucose induced apoptosis of cells in a time and concentration‐dependent manner. Apoptosis was guided by the mitochondrial permeability transition pore (mPTP)/ROS/Caspase‐3 pathway and was dependent on AE2. Indeed, glucose upregulated the expression as well as the activity of AE2, as evidenced by an increased intracellular Cl^−^ concentration. Pharmacological inhibition or silencing of the expression of AE2 protected cells from apoptosis.^111^

Turi and collaborators have reported that the expression of AE2 in rat airway epithelial cells is regulated by OS, and this regulation seems to be mediated by transcription factor AP‐1, a specific protein able to respond to elevated intracellular concentrations of ROS, including H_2_O_2_.^112^ The transport of superoxide radicals *via* AE2 raises interesting questions with regard to the regulation of AE2 in the lung. Since superoxide radicals can contribute to oxidant lung injury, the increase of expression of AE2 following OS exposure should provide increased transport of O_2_^·−^ out of the cell, thus limiting the oxidative damage. Accordingly, AE2 has been demonstrated to protect cell viability in a model of lung reperfusion injury.^113^ However, other studies have shown a decreased injury when the function of the AE2 exchanger was blocked.^114^ Therefore, the expression of AE2 and its function can be altered by OS, but it remains unclear how such alterations occur and, ultimately, whether this molecular entity can protect or rather injure cells during periods of elevated OS.

## 3 THE SLC26 FAMILY

The SLC26 family of multifunctional ion transporters and channels comprises 11 genes (*SLC26A1‐11*) of which 10 are protein coding and one (*SLC26A10*) is a pseudogene. With the exception of *SLC26A5*, which encodes for the outer hair cells motor protein prestin, these genes encode multifunctional anion exchangers, of which SLC26A7, A9 and A11 can also operate as uncoupled ion transporters in a channel‐like mode. The anion exchangers of this family can accept divalent as well as monovalent anions and generally show versatile substrate selectivity, being able to exchange chloride for inorganic anions such as bicarbonate, hydroxyl, sulphate and iodide, or small organic anions such as formate and oxalate. SLC26A3, A4, A6, A7, A9, and A11 can operate in Cl^−^/HCO_3_^−^ exchange mode, while SLC26A1 and A2 are selective sulphate transporters.^115^, ^116^, ^117^

The SLC26 family was discovered more recently and is less well characterized compared with SLC4. SLC26 members are highly hydrophobic, large (700‐1,000 amino acids) proteins sharing relatively low (21%‐43%) amino acid identity with the members of the same family. The structure of mammalian SLC26 transporters has long been elusive. Structures of the murine Slc26a9,^118^ two bacterial homologues—a bicarbonate transporter from cyanobacteria (BicA)^119^ and a proton‐coupled fumarate transporter from the bacterium *Deinococcus geothermalis* (SLC26Dg),^120^ and a plant homologue of SLC26—the vacuolar H^+^/SO_4_ ^2^ symporter SULTR4;1 from *Arabidopsis thaliana* ^121^ were solved recently by X‐ray crystallography and cryo‐electron microscopy. These structures show a shared architecture consisting of 14 transmembrane α‐helices organized in two intertwined inverted repeats of seven transmembrane segments each and a large C‐terminal cytosolic domain referred to as the sulphate transporter and anti‐sigma factor antagonist (STAS) domain. It is widely accepted that mammalian SLC26 members and their lower organism counterparts act as functional homodimers, although the dimerization mechanism was not unequivocally determined and probably differs among family members. Two swapped STAS domains have been determined as major determinants of the homodimeric assembly of murine Slc26a9,^122^ *Arabidopsis thaliana* SULTR4;1^121^ and BicA from *Synechocystis* sp,^119^ while the STAS domain is not a requisite for SLC26Dg dimerization, which indeed relies on contacts between the transmembrane domain and is centred on TM14.^123^

Some of the members of the SLC26 family show restricted tissue distribution, whereas other isoforms are more broadly expressed and orchestrate the ion transport across epithelia such as the intestine and kidney.^124^, ^125^ Pathogenic sequence alterations in *SLC26* genes cause inherited diseases, including diastrophic dysplasia (phenotype MIM number 222600) and other osteochondrodysplastic syndromes (SLC26A2), secretory chloride diarrhoea (SLC26A3, phenotype MIM number 214700), Pendred syndrome (SLC26A4, phenotype MIM number 274600) and non‐syndromic deafness (SLC26A4 and A5, phenotype MIM numbers 600791 and 613865, respectively), calcium oxalate nephrolithiasis (SLC26A1 and A6, phenotype MIM number 167030), and spermatogenic failure (SLC26A8, phenotype MIM number 606766). SLC26A3, A4, A6, A8 and A9 physically and/or functionally interact with the CFTR chloride channel and regulate the CFTR activity or are regulated by CFTR.^126^, ^127^, ^128^, ^129^

The information regarding the impact of OS on SLC26 function is sparse, and will be reviewed in the following and in Table 2.

**TABLE 2:** Link between SLC26 members and oxidative stress (OS). Anion exchangers of this family are all Na^+^‐independent

Transport mode	Isoform	Experimental model	Findings	References
Anion exchanger	SLC26A1	No information available		
	SLC26A2	No information available		
	SLC26A3	CaCo‐2 cells exposed to H_2_O_2_	Reduction of DIDS‐sensitive ^36^Cl^−^ uptake	135
		Tissue from patients with active ulcerative colitis	Decreased transcript levels in the intestine	136
	SLC26A4	Knockout mouse	Increased OS in the *stria vascularis*	142
		Pendred syndrome thyroid	Increased OS	145
		Rats exposed to potassium bromate	Decreased transcript levels in the kidney	146
		Mouse models of OS	Increased transcript levels in the cochlea	144
		IL‐4/IL‐13‐stimulated, SLC26A4‐mediated SCN^−^ secretion	OS, inflammation, necrosis	151
**—**	SLC26A5	Placenta of smoking mothers	Increased transcript levels	155
Anion exchanger	SLC26A6	CaCo‐2 cells exposed to H_2_O_2_	Reduction of DIDS‐sensitive ^36^Cl^−^ uptake	135
		Rat proximal tubule cells, rat kidney	Oxalate‐induced OS, cell injury, apoptosis	162
Anion exchanger/channel	SLC26A7	No information available		
Anion exchanger	SLC26A8	No information available		
Anion exchanger/channel	SLC26A9	No information available		
Anion exchanger/channel	SLC26A11	No information available		

### 3.1 SLC26A3/DRA

SLC26A3/DRA was initially identified as a candidate tumour suppressor gene that was downregulated in adenoma (hence the alias DRA).^130^ Later, SLC26A3 was identified by positional cloning as the gene mutated in recessive congenital chloride‐losing diarrhoea.^131^ This transporter is expressed on the apical membrane of epithelial cells in the intestine and is particularly abundant in the colon and duodenum. SLC26A3 functions as a Cl^−^/HCO_3_^−^ exchanger in tandem with NHE3, which mediates a Na^+^/H^+^ exchange, to generate an electroneutral NaCl reabsorption across the intestinal mucosa, or gives rise to HCO_3_^−^ secretion in the absence of NHE3.^117^

#### 3.1.1 SLC26A3/DRA and oxidative stress

Oxidative stress plays an essential role in the pathogenesis and progression of inflammatory bowel disease (IBD).^132^ It is well known that SLC26A3 expression is reduced in animal models of intestinal inflammation as well as in patients with ulcerative colitis (UC), an effect that was attributed to inhibition of gene transcription by proinflammatory cytokines.^133^, ^134^ Interestingly, H_2_O_2_ inhibited the SLC26A3 and SLC26A6‐mediated Cl^−^/OH^−^(HCO_3_^−^) exchange activity in Caco‐2 cells independently of the prostaglandin/COX‐dependent pathway, but occurring *via* a signalling cascade that involved the activation of the Src kinase Fyn, PI3K, PLC*γ* 1 and the Ca^2+^‐dependent PKC*α*.^135^ These authors concluded that phosphorylation of the anion exchangers or regulatory proteins, rather than modification of their plasma membrane trafficking, might be involved in modulation of their activity.

In a recent study, the gene expression of SLC26A3 was decreased in the colonic mucosa from patients with active UC compared with patient in remission and non‐inflamed donors. In parallel, SOD2 was significantly upregulated in the colonic mucosa from patients with active UC compared with controls, thus denoting an increased OS, and SOD2 levels correlated with severe histological activity in the inflamed mucosa.^136^ Together, these findings suggest that OS might contribute to inhibition of expression and function of SLC26A3 in the context of IBD, thus aggravating fluid loss and stool acidification consequent to lack of NaCl reabsorption and HCO_3_^−^ secretion.

### 3.2 SLC26A4/pendrin

SLC26A4/pendrin is an electroneutral Cl^−^/anion exchanger abundantly expressed on the apical membrane of distinct epithelial cells of the *stria vascularis* and endolymphatic duct and sac of the inner ear, as well as in the thyroid and kidney. In the inner ear, pendrin drives HCO_3_^−^ secretion and Cl^−^ reabsorption and controls the endolymphatic pH and volume. In the thyroid, pendrin participates in the iodide flux into the thyroid follicle, possibly via its I^−^/Cl^−^ exchange activity. In addition, pendrin expression is upregulated in the airways and oesophageal mucosa by the pro‐inflammatory cytokines IL‐4/IL‐13 via a STAT6‐mediated pathway.^137^, ^138^ Loss or reduction of function of pendrin consequent to gene mutation causes autosomal recessive forms of non‐syndromic as well as syndromic sensorineural hearing loss, ie, DFNB4 and Pendred syndrome, of which a malformation of the inner ear called enlarged vestibular aqueduct (EVA) with or without cochlear incomplete partition type 2 is the main radiological finding. Vestibular dysfunction is also observed in a fraction of patients. In Pendred syndrome, deafness is associated with a partial iodide organification defect in the thyroid that may lead to subclinical or overt hypothyroidism and goiter.^139^, ^140^ In the cortical collecting duct and connecting tubule of the kidney nephron, pendrin is expressed on the apical membrane of β and non‐α, non‐β intercalated cells, mediates Cl^−^ reabsorption and HCO_3_^−^ excretion and controls the systemic electrolyte, vascular volume and acid‐base homoeostasis by working in concert with other ion absorbing transport systems, including the sodium chloride cotransporter NCC and the epithelial sodium channel ENaC.^141^

#### 3.2.1 SLC26A4/pendrin and oxidative stress

A possible link between OS and pendrin expression and function in the inner ear was firstly reported by Wangemann and collaborators, who described hyperpigmentation of the *stria vascularis* in pendrin‐knockout mice. Hyperpigmentation of the *stria vascularis* was linked to an increased melanin production in the strial intermediate cells, which are in charge of detoxification of free radicals produced by the metabolically active strial marginal cells. The authors proposed a model where lack of bicarbonate secretion in the endolymph due to the absence of pendrin, with consequent increase of bicarbonate concentration and pH in the intra‐strial fluid, may have led to inhibition of cysteine uptake and glutathione synthesis in the intermediate cells and free radical damage of these cells. Oxidative stress in the intermediate cells was proposed to be responsible for the reduced protein expression of KCNJ10/Kir4.1, an inwardly rectifying K^+^ channel essential for the generation and maintenance of the endochoclear potential and proper hearing function.^142^ In this context, pH control via pendrin would exert a protective role against OS. Accordingly, elevated amounts of oxidized and nitrated proteins were found in the *stria vascularis* of pendrin‐knockout mice, and OS reduced Kcnj10 expression in a heterologous system.^143^ These findings underscore the physio‐pathological relevance of OS, leading to failure of KCNJ10 expression, lack of endochoclear potential and deafness in mouse models and possibly in patients with pendrin‐related hearing loss.

A recent study has evidenced a significant increase in the Slc26a4 transcript levels in the cochlea of three distinct mouse models of age‐related hearing loss linked to chronic OS, represented by mice subjected to intermitted hypoxia alone, high‐fat diet plus galactose injection and intermitted hypoxia combined with high‐fat diet and galactose injection for 12 weeks, respectively. These changes were paralleled by an increase in the hearing threshold and morphological damage of cochlear hair cells.^144^ Although the pathophysiological significance of the altered Slc26a4 transcript levels in these mouse models remains unclear, this study suggests that pendrin transcriptional regulation might respond to OS with compensatory changes.

In the thyroid, iodide reaches the follicular lumen *via* pendrin and is subsequently incorporated (organified) into thyroglobulin (Tg) by the thyroid peroxidase (TPO), which requires H_2_O_2_ as cofactor. H_2_O_2_ is generated by dual oxidases (DUOXs). In a normal thyroid, TPO and DUOX expression is restricted to the apical membrane of thyrocytes or confined within Caveolin‐1‐positive intracellular vesicles. Similar to what was observed in the inner ear of pendrin‐knockout mice, OS was augmented in the thyroid of a patient with Pendred syndrome, as evidenced by greatly increased lipid peroxidation as well as catalase and peroxiredoxin PRDX5 expression. In parallel, TPO and DUOX showed greatly enhanced expression, lost their normal apical distribution and were mislocalized within the cytosol, while the expression of Caveolin‐1 was absent. These authors linked the increased OS in the thyroid tissue with aberrant, intracellular thyroid hormone biosynthesis, a process that would require H_2_O_2_ production within the cytosol.^145^ Although the mechanism by which TPO and DUOX were mislocalized remained unexplained, these findings underscore the importance of proper pendrin activity in protecting from dramatic OS‐related pathological changes in the thyroid.

Pendrin transcript was found downregulated (−4.6 and −6.0 fold after 52 and 100 weeks, respectively) in the kidney from F344 male rats exposed to 400 ppm (high) potassium bromate in drinking water. Potassium bromate is a nephrotoxic compound and a kidney and thyroid carcinogen, can induce deafness and promotes OS, as evidenced by de‐regulation of OS‐related genes.^146^ Although the mechanism leading to decreased pendrin mRNA levels remained unexplained, this study suggests that pro‐oxidant compounds might suppress pendrin transcription in the kidney and perhaps other organs, including the inner ear. Further studies are needed to verify this hypothesis.

Similarly to thyroid, the airway and lung epithelium has the ability to generate H_2_O_2_ at the apical membrane through DUOX1 and DUOX2. H_2_O_2_ serves to oxidize luminal SCN^−^ to OSCN^−^ (hypothiocyanite) in a reaction catalyzed by lactoperoxidase and other peroxidases. Both anions are pro‐oxidant species, have an antimicrobial role and play an important function in the innate mucosal defense. Pendrin is a major determinant of the IL‐4/IL‐13‐stimulated SCN^−^ secretion at the luminal surface of human bronchial epithelial cells and lung.^147^, ^148^, ^149^ When upregulated by ILs in the context of airway inflammation and allergic asthma, SCN^−^ secretion may lead to increased OSCN^−^ production. OSCN^−^ in low doses can be sensed by the OS‐sensitive PKA, which dimerizes and activates NF‐κB, a transcription factor critical for inflammatory responses. High doses of OSCN^−^ can induce necrosis of epithelial cells, further triggering inflammation.^149^ The implications of these findings are that (i) a defective SCN^−^ secretion *via* pendrin might be linked to increased bacterial colonization of the airway epithelium^147^ and (ii) inhibition of pendrin activity^150^ or peroxidase‐dependent conversion of SCN^−^ to OSCN^−^ ^151^ might be beneficial in treating asthma and other chronic inflammatory airway conditions. These studies highlight how ion transport, OS and inflammation are tightly interconnected and their dysregulation might profoundly affect mucosal function and health.

### 3.3 SLC26A5/prestin

SLC26A5/prestin is the transmembrane sensor‐motor protein of the outer air cells (OHC) of the mammalian organ of Corti and enables changes in length of these cells depending on the frequency of a sound stimulus, which is the basis of the mechanism of cochlear amplification of sounds.^152^ Although prestin shares high homology with other members of the SLC26 family of proteins including pendrin and DRA, it does not transport ions across the plasma membrane, but instead changes its structure by voltage‐dependent translocation of anions within the molecule itself.^153^ Targeted deletion of prestin exons 3‐7 in mice resulted in loss of OHC electromotility in vitro and a 40‐60 dB loss of cochlear sensitivity in vivo.^154^ A limited number of *SLC26A5* mutations has been identified in patients with hearing loss; one of these mutations was linked to autosomal recessive deafness DFNB61 but later was found non‐pathogenic.^9^ Due to the limited number of patients, whether any of the identified SLC26A5 mutations are true causes of hearing loss remains unclear.

#### 3.3.1 SLC26A5/prestin and oxidative stress

Katbamna et al have shown that prenatal smoke exposure (≥10 cigarettes/d) leads to significant reductions in the cochlear response amplitudes and auditory brainstem recording (ABR) wave latencies in newborns. These alterations in the auditory function were paralleled by a dysregulation of the expression of placental genes, including SLC26A5, which was upregulated 8.95 fold, again suggesting an adaptive change. Also, several OS pathway genes were found dysregulated. The authors suggested that placental gene expression might be a good surrogate of foetal gene expression.^155^ The study raises the possibility that prenatal exposure to OS might damage the hearing function in the newborn by targeting genes involved in regulating the hearing function.

Oxidative stress following production of ROS is a hallmark of noise‐induced hearing loss and mainly targets OHC.^156^ Prestin was used to selectively target ROS‐scavenging nanoparticles to OHC in a guinea pig model of noise‐induced hearing loss. This innovative strategy allowed for preservation of the morphological integrity of OHC and led to significant improvement of the hearing function, denoting that protection of OHC and their delicate molecular machinery from OS is crucial in the prevention and treatment of noise‐induced hearing loss.^157^

### 3.4 SLC26A6/PAT1/CFEX

SLC26A6 putative anion transporter 1 (PAT1) and chloride‐formate exchanger (CFEX) was cloned based on the homology to the genes encoding SLC26A3 and SLC26A4, was found most abundantly expressed in the kidney and pancreas,^158^ but was also detected in other tissues, including the intestine. SLC26A6 can work in Cl^−^/HCO_3_^−^, Cl^−^/OH^−^, chloride/oxalate and chloride/formate exchange modes.^159^ Mutant mice lacking Slc26a6 develop calcium oxalate urolithiasis, have significant hyperoxaluria and elevation in plasma oxalate concentration, both resulting from a defect in intestinal oxalate excretion, which leads to an enhanced net absorption of oxalate.^160^ In the human pancreas and intestine, SLC26A6 is co‐expressed with SLC26A3 and CFTR and participates in chloride‐dependent HCO_3_^−^ secretion by acting synergistically with CFTR; in addition, SLC26A6 mediates oxalate excretion in the intestine and kidney proximal tubule and Cl^−^/HCO_3_^−^ exchange in the myocardium. These findings imply a role of SLC26A6 in the development of intestinal and pancreatic diseases, nephrolithiasis and arrhythmia.^161^

#### 3.4.1 SLC26A6/PAT1/CFEX and oxidative stress

In normal rat proximal tubular epithelial (NRK‐52E) cells, SLC26A6 expression correlated with oxalate‐induced cell injury, apoptosis, crystal adhesion, ROS formation and lipid peroxidation. Accordingly, selective attenuation of SLC26A6 expression in the kidney of rats with lentivirus‐transfected siRNAs decreased SOD generation, cell apoptosis and crystal formation.^162^ This study highlights that SLC26A6‐mediated oxalate excretion in the kidney might be a determinant of stone formation as well as OS‐induced cell injury and identifies SLC26A6 as a target of potential protective therapeutic approaches.

## 4 CONCLUDING REMARKS

Several studies have investigated in detail the impact of OS on some transporters of the SLC4 family in different experimental conditions (Table 1). In particular, great attention has been reserved to SLC4A1. In this regard, human erythrocytes exposed to various oxidizing agents or extracellular pH variations have shown both a reduction in transport efficiency and changes in the structural state of SLC4A1. Instead, in ex vivo or in vitro models of inflammation, diabetes mellitus and ageing, the SLC4A1 ion transport was found accelerated. While the reasons of this apparent discrepancy are only partly elucidated, it becomes increasingly obvious that the activity of SLC4A1 is sensitive to OS and increases or decreases in ion transport efficiency most likely reflects the formation and/or the amount of glycated haemoglobin and methaemoglobin, as well as the integrity of the lipid bilayer ad the intricate intracellular network of interacting proteins (Figure 3).

The potential impact of OS on the expression and activity of SLC26 family members was less extensively studied compared with SLC4A1. For several SLC26 members, there is no information available in this context. The current knowledge denotes a dual role of these transporters in being potential targets as well as determinants of OS (Figure 3). For example, function and possibly expression of SLC26A3 and SLC26A6 can be reduced during OS. Similarly, SLC26A4 and SLC26A5 gene expression appeared dysregulated in OS. In turn, lack of expression of SLC26A4 was associated with increased OS in the human thyroid and mouse cochlea. At the same time, SLC26A4 upregulation in the context of inflammation and SLC26A6 appeared aggravating or mediating OS (Table 2). More studies are needed to explain how SLC26 members sense, respond to or determine OS. Particularly intriguing is the hypothesis of a possible dysfunction of SLC26A4 and SLC26A5 mediated by OS in noise‐induced and/or age‐related hearing loss.

Further research should establish whether and how members of the SLC4 and SLC26 family of proteins can be safely and effectively targeted by antioxidants in the prevention and treatment of OS‐related conditions, including inflammation, metabolic dysfunctions, hearing loss and ageing.

## CONFLICTS OF INTEREST

The authors declare no conflict of interest.

## AUTHOR CONTRIBUTIONS

All authors contributed to performing the literature search and writing the manuscript. All authors have read and agreed to the final version of the manuscript.
